# m6Acorr: an online tool for the correction and comparison of m^6^A methylation profiles

**DOI:** 10.1186/s12859-020-3380-6

**Published:** 2020-01-29

**Authors:** Jianwei Li, Yan Huang, Qinghua Cui, Yuan Zhou

**Affiliations:** 1 Institute of Computational Medicine, School of Artificial Intelligence, Hebei University of Technology, Tianjin, China; 2 Department of Biomedical Informatics, School of Basic Medical Sciences, Center for Noncoding RNA Medicine, Peking University, Beijing, China

## Abstract

**Background:**

The analysis and comparison of RNA m^6^A methylation profiles have become increasingly important for understanding the post-transcriptional regulations of gene expression. However, current m^6^A profiles in public databases are not readily intercomparable, where heterogeneous profiles from the same experimental report but different cell types showed unwanted high correlations.

**Results:**

Several normalizing or correcting methods were tested to remove such laboratory bias. And m6Acorr, an effective pipeline for correcting m^6^A profiles, was presented on the basis of quantile normalization and empirical Bayes batch regression method. m6Acorr could efficiently correct laboratory bias in the simulated dataset and real m^6^A profiles in public databases. The preservation of biological signals was examined after correction, and m6Acorr was found to better preserve differential methylation signals, m^6^A regulated targets, and m^6^A-related biological features than alternative methods. Finally, the m6Acorr server was established. This server could eliminate the potential laboratory bias in m^6^A methylation profiles and perform profile–profile comparisons and functional analysis of hyper- (hypo-) methylated genes based on corrected methylation profiles.

**Conclusion:**

m6Acorr was established to correct the existing laboratory bias in RNA m^6^A methylation profiles and perform profile comparisons on the corrected datasets. The m6Acorr server is available at http://www.rnanut.net/m6Acorr. A stand-alone version with the correction function is also available in GitHub at https://github.com/emersON106/m6Acorr.

## Background

RNA m^6^A methylation, one of the most common RNA modifications [1], is crucial for the post-transcriptional regulations of gene expression processes, such as mRNA degradation, translation, and alternative splicing [2, 3]. Recent studies also suggest its critical roles in various biological processes, including stem cell fate decision, oncogenesis, and long-term memory consolidation [4–6].

In line with its emerging functional importance, current specialized databases, such as MeT-DB and RMBase, have accumulated a sizable amount of m^6^A methylation profiles [7, 8]. In one methylation profile, the relative methylation level of each gene can be described as an enrichment score comparing the methylated read counts (m^6^A-IP library) to the total read counts (input library). Ideally, the hyper- (hypo-) methylated genes of each sample can be easily determined on the basis of the enrichment score if the methylation profiles are readily intercomparable.

**Fig. 1:**
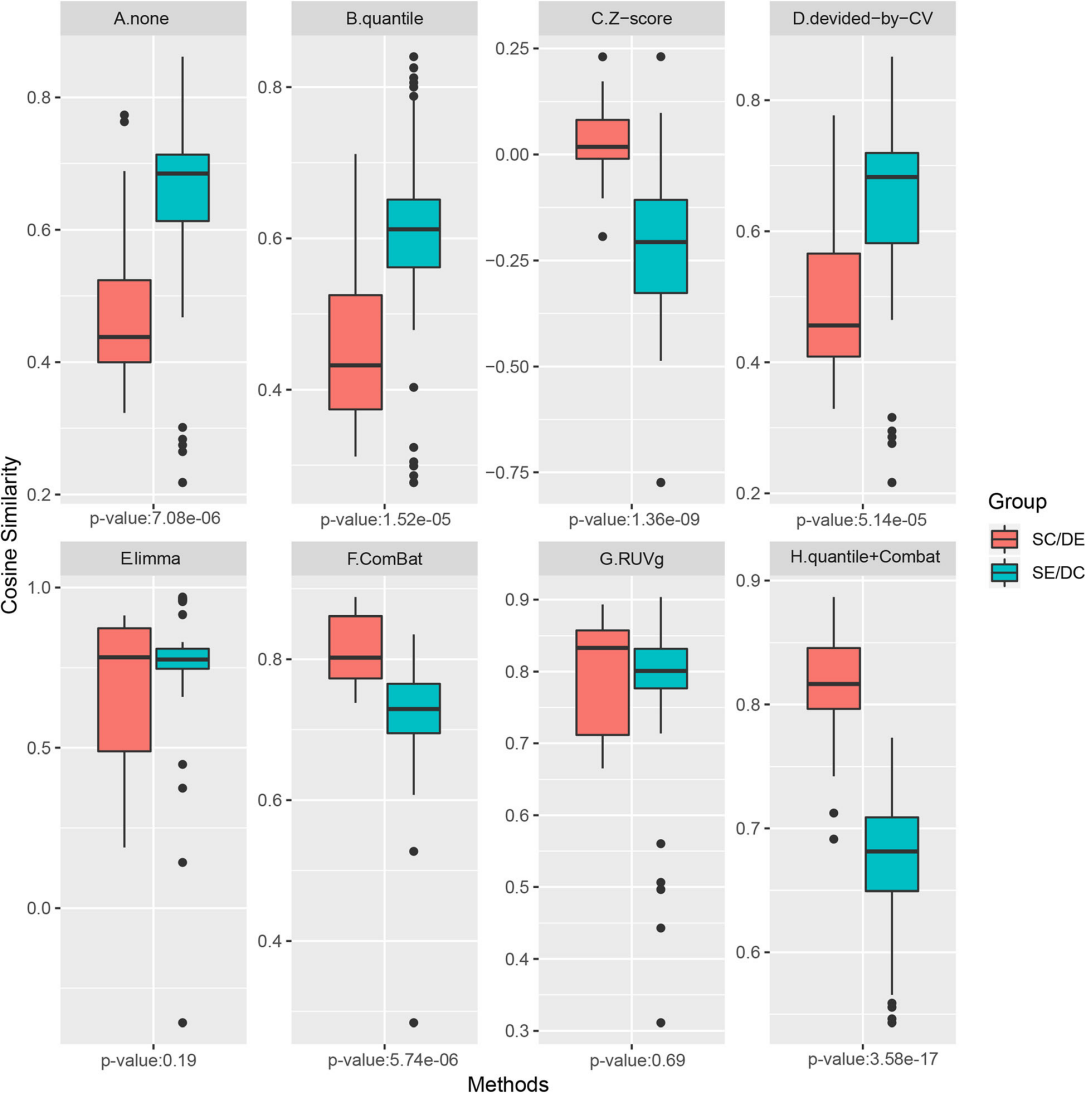
Comparison of intragroup correlations between the SE/DC and SC/DE groups in the human methylation dataset. SE/DC, same experiment but different cell types; SC/DE, same cell type across different experiments. Intuitively, methylation profiles with low bias should have significantly higher correlation in SC/DE group than that in the SE/DC group, which can be achieved by combining the ComBat method and quantile normalization. *P*-values were obtained by t-test

However, preliminary analysis of MeT-DB data failed to validate the intercomparability of current methylation profiles. Notably, all methylation profiles from MeT-DB were generated by the same computational pipeline to remove algorithm discrepancies. Therefore, intuitively, in the absence of other prominent biases, the methylation profiles of the same cell or tissue type should show prominent similarity relative to those from the same experimental report. However, the current results on the human dataset indicated the opposite: the methylation profiles from the same experiment but different cell types showed unwanted high correlations, whereas the correlations among the same cell type profiles across different experiments were only moderate (Fig. 1a). This tendency can also be observed in the mouse dataset (Additional file 1: Figure S1A). These results highlighted the serious but previously ignored laboratory bias in current m^6^A methylation profiles. Several well-known normalization and batch regression methods [9] for gene expression profile correction were attempted to correct such bias, and a valid correction pipeline was finally established. This pipeline has been made available as a web server named m6Acorr, wherein users could correct their m^6^A methylation profiles and perform comparative analysis based on the corrected profiles.

## Implementation

### Real-world m^6^A profile dataset

After removing methylation profiles with small amounts of genes (e.g., methylation profiles of small RNAs), 36 human samples from seven experiments and 13 cell/tissue types were obtained from MeT-DB v2.0. The samples were then divided into two groups: SE/DC (same experiment but different cell types) and SC/DE groups (same cell type across different experiments). Cosine correlation was used to demonstrate the similarity between the two methylation profiles within the same group to avoid artifacts resulting from zero scores. Finally, intra-group similarities based on the two grouping criteria were compared to check the potential laboratory bias.

### Simulated dataset

R (v3.6.1) package Splatter (v1.10.0) was utilized to simulate RNA m^6^A methylation profiles with laboratory bias. Splatter gathers the parameters that reflect the distribution of real data and generates simulative data with these estimated parameters. *splatEstimate* and *splatSimulate* functions were respectively used to acquire parameters and generate an artificial dataset with laboratory bias. Two parameters, namely, batch.facLoc and batch.facScale, which respectively reflect location and scale for the batch effect factor log-normal distribution, were optimized through grid search to match the distribution of the m6A profiles from the real-world dataset (Additional file 1: Table S1). Finally, batch.facLoc and batch.facScale were set to 0.3 and 0.2 respectively which were closest to the real world. An extreme case that set batch.facLoc and batch.facScale to 0.4 was also tested and consistent results could be achieved (SE/DC under SC/DE after correction, *P* = 3.11e-17). Finally, an artificial dataset with 20 profiles, including four batches (experiments) and two cell types, was obtained to test the performance of m6Acorr.

### Methylation profile correction method

The methylation correction pipeline is closely related to popular gene expression profile correction methods. Seven popular methods for normalizing and/or correcting RNA-seq and microarray data were tested to correct observed laboratory bias in the methylation profiles. (1) Quantile normalization. According to the standard setting [10], each column of the m^6^A profiles matrix *X* was first sorted to obtain *X*_*ordered*_, and then each row of *X*_*ordered*_ was replaced with the average of the row. Finally, the matrix *X* was replaced by the corresponding average value based on the *X*_*ordered*_ and acquired *X*_*quantile*_. Q1, Q2, and Q3 quantiles were also attempted to replace the average (i.e., the standard setting); the standard setting performed slightly better than using alternative settings (Additional file 1: Figure S2). (2) Z-score normalization among samples from the same experiment. The Z-score can be defined as follows:

1 $$ Z=\frac{x-\mu }{\sigma } $$

where x is the enrichment score of the gene; *μ* represents the average enrichment score of one experiment; and *σ* is the standard deviation of the sample. (3) Division of the per experiment coefficient of variation (CV). CV can be described by the following equation:

${C}_v=\frac{\sigma }{\mu }$ (2)where *σ* is the standard deviation of the sample space, and *μ* represents the sample average. (4) The empirical Bayes-based batch regression method from limma package (v3.42.0) [11] was performed with the default parameters. (5) The empirical Bayes-based batch regression method from SVA package (v3.34.0), which is also known as the ComBat method [12], was performed with the default parameters. (6) The RUVg method from RUVseq package (v1.20.0), which removes the batch effect according to the control genes [13], was performed. In this study, the top 10% genes showing the most constant expression in the GTEx transcriptome atlas were selected as the control genes [14]. (7) Considering the possible differences between the profiles, these genes were adjusted to the same distribution with quantile normalization to coordinate with the Combat model for correction. The combined pipeline is finally adopted in m6Acorr, which is closely related to the recommended pipeline for gene expression correction [9].

### Analysis of corrected methylation profiles

The m6Acorr server is available at http://www.rnanut.net/m6Acorr. In addition to profile correction, the m6Acorr server also provides the comparative analysis functions of the corrected profiles. After correction, hyper- (hypo-) methylated genes could be obtained on the basis of the Z-score comparison of user-provided samples to the MeT-DB samples. The functional enrichment analysis of the hyper- (hypo-) methylated genes is also enabled by investigating the enrichment of gene sets curated from (1) WikiPathways pathway gene sets [15]; (2) MSigDB hallmark gene sets [16]; (3) RNA-binding protein target genes from ENCODE project [17]; and (4) miRNA target genes from miRTarBase using the hypergeometric test [18]. Finally, a stand-alone version with the correction function is also available in GitHub at https://github.com/emersON106/m6Acorr.

## Results

As previously described in the Background Section, the high correlation among the samples from the same experiment but different cell types (i.e. the SE/DC group) suggested serious laboratory bias. This result indicates that heterogeneous profiles show unwanted higher similarity compared with those from the same cell type across different experiments (i.e. the SC/DE group) (SE/DC over SC/DE, t-test, *P* = 7.08e-06; Fig. 1 a). Several normalization methods have been applied, and only Z-score normalization partially reversed this biased correlation (Fig. 1b–d). Several popular batch-regression methods were further applied because normalization methods were not intended for experimental batch bias correction. Among these methods, the ComBat method successfully reversed the high correlation in the SE/DC group (SE/DC under SC/DE, *P* = 5.74e-06; Fig. 1f). Finally, by combining ComBat and quantile normalization, the unwanted high correlation in the SE/DC group was prominently reversed, implying effective bias elimination (SE/DC under SC/DE, *P* = 3.58e-17; Fig. 1h). The mouse dataset from MeT-DB V2.0 and the simulated data for independent verification of the correction pipeline were further adopted. Similar results were observed for the mouse dataset (Additional file 1: Figure S1), where the unwanted high correlation (SE/DC over SC/DE, *P* = 2.44e-08) was successfully reversed (SE/DC under SC/DE, *P* = 9.52e-14). Such a tendency reversal on the mouse dataset verified the validity of the “quantile + Combat” correction pipeline for the real-world dataset. In addition, considering the limited coverage of current m^6^A profiles, the correction pipeline was applied to an artificial simulated dataset. Notably, the “quantile + Combat” correction pipeline on this simulated dataset also exhibited competitive efficiency (SE/DC under SC/DE, *P* = 1.99e-17 after correction; Additional file 1: Figure S3). Therefore, the “quantile + Combat” combination pipeline, which was named m6Acorr hereafter, was selected for m^6^A methylation profile correction.

**Fig. 2:**
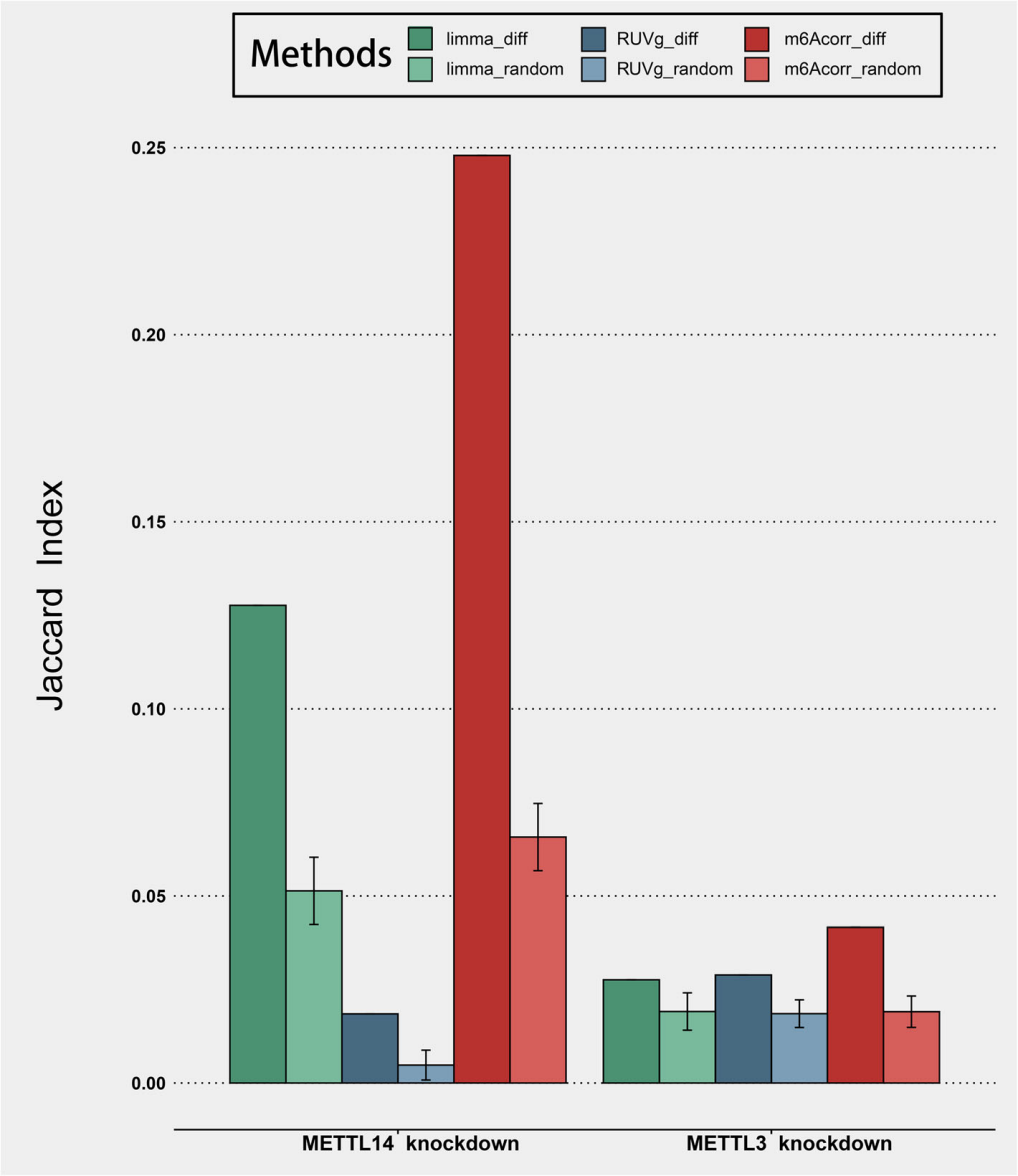
Jaccard index depicting the shared fraction between the differentially methylated genes identified before and after correction by three methods. Diff: the top 20% differentially methylated genes; random: randomly selected same amount of genes (repeated 100 times, error bar showing the standard error)

**Fig. 3:**
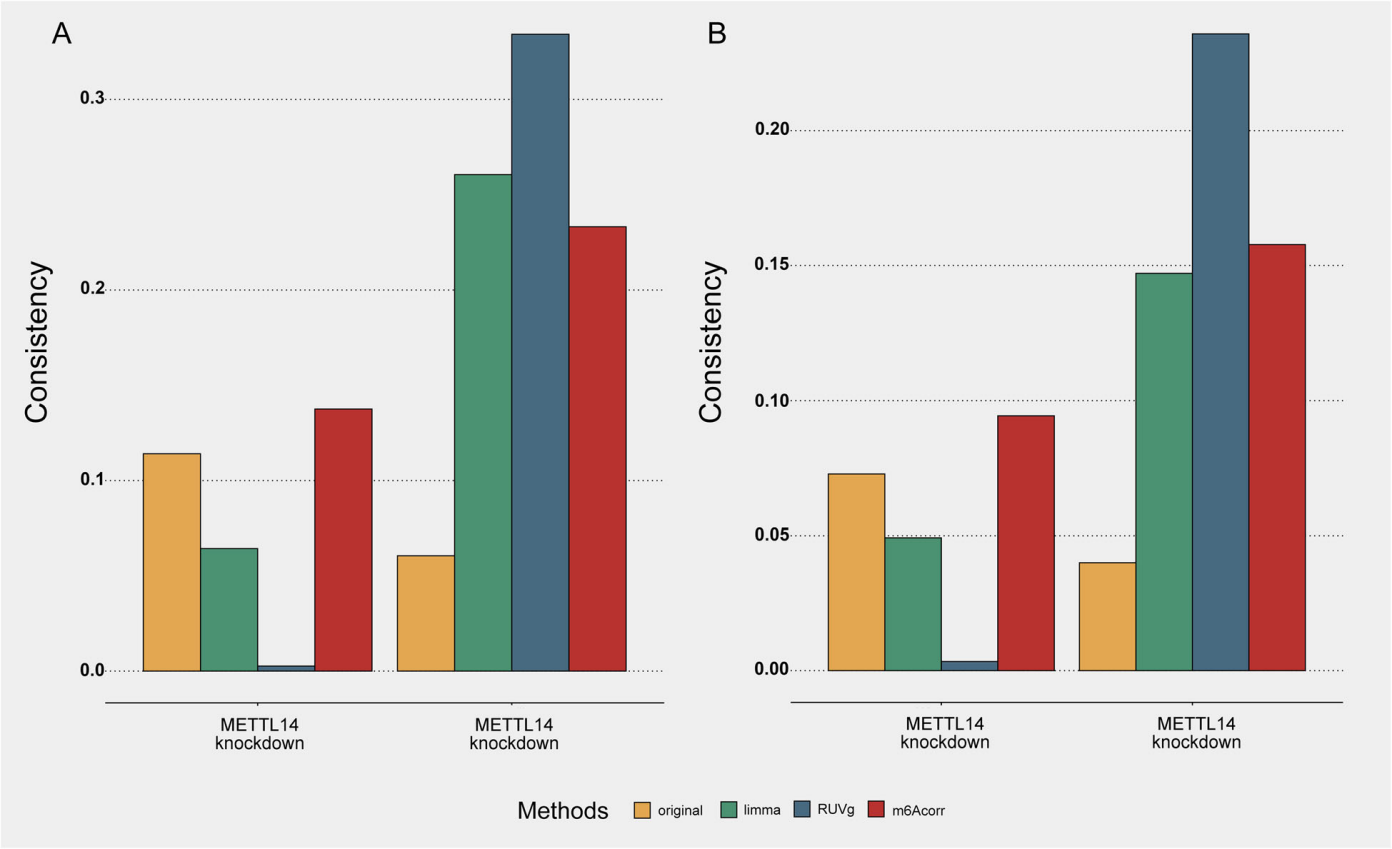
Consistency of differentially methylated genes (before and after corrections) with functional m^6^A target genes. **a**. The consistency with the m^6^A target genes whose translation efficiency is significantly reduced after *METTL3* or *METTL14* knockdown. **b**. The consistency with the m^6^A target genes whose mRNA stability is significantly increased after *METTL3* or *METTL14* knockdown

Although m6Acorr could efficiently reduce laboratory bias, one prominent concern is the elimination of biological signals after correction. The retention of differential methylation signals was first examined to further check if the m6Acorr pipeline would disturb biological signals and thus obstruct downstream applications. To this end, two representative pairs of profiles in MeT-DB V2.0, which investigate the alteration in m^6^A methylation after the knockdown of methyltransferases *METTL3* and *METTL14* (the enzymes catalyzing m^6^A methylation), were selected (p007_HeLa1_ctrl with p007_HeLa1_KO_M14, and p007_HeLa2_ctrl with p007_HeLa2_KO_M3). Note also that these profiles were independent from the above assessment of correction pipelines because they were derived from the m6A enzyme mutant cells rather than wild-type cells. The shared differentially methylated genes before and after correction were compared by calculating the Jaccard index between the top 20% differentially methylated genes. As shown in Fig. 2, m6Acorr exhibited better preservation of differentially methylated genes than the alternative methods. In addition, the fraction of shared differentially methylated genes could not be achieved by randomly selected genes. Given that the fraction of preserved differentially methylated genes was only moderate, the methods were examined by checking if the differentially methylated genes identified after correction could show good consistency with the functional m^6^A target genes [2, 19]. Two typical classes of functional m^6^A target genes were considered. The first class is the genes whose translational efficiency is intensively regulated by m^6^A modification. These genes showed remarkable decreases in translational efficiency after *METTL3* or *METTL14* knockdown as recorded in the GEO dataset GSE63591. The second class is the genes whose mRNA stability is intensively regulated by m^6^A modification. These genes showed significant increases in mRNA stability after *METTL3* or *METTL14* knockdown as recorded in the GEO dataset GSE49339. The comparison results are summarized in Fig. 3. Interestingly, differentially methylated genes identified after correction showed good consistency with either class of functional m^6^A target genes, even when compared with the differentially methylated genes identified from the uncorrected methylation profiles. These results indicated that methylation profile correction by m6Acorr is also helpful for finding the important functional targets of m^6^A regulation. Finally, the comparative analysis of the biological features of frequently and occasionally methylated genes was performed using the corrected methylation profiles. Previous comparative analysis using the uncorrected methylation profiles indicated that the overall m^6^A methylation breadth across samples was correlated with gene importance-related features, including dN/dS ratio, tissue expression specificity, and PPI network degree [20]. Notably, such significant correlations were retained after correction (Additional file 1: Figure S4), indicating the preservation of biological signatures.

## Discussion

In the above section, we have demonstrated the effectiveness of the ‘normalization + correction’ pipeline for m^6^A methylation profile correction. The pipeline adopts well-known methods for gene expression profile correction [9]. However, the suitability and effectiveness of such methods on m^6^A methylation profile correction is not naturally guaranteed. In fact, at the start point, to which extent laboratory bias exists in m^6^A methylation profiles has not been systematically explored. As for the intuitive assumption according to the principle of m^6^A profiling technique (i.e. MeRIP-seq) [7, 8], since the methylation levels are derived by comparing the methylated read counts against the total read counts from the same sample, the laboratory bias should be canceled out during such intra-sample comparison. But as what we have shown above, the laboratory bias turns out to be quite serious, and on the other hand, not all of the well-known methods work well for the methylation profile correction. Therefore, the novelty of this study is focused on why and how the correction pipeline should be applied to the m^6^A methylation profiles.

**Fig. 4:**
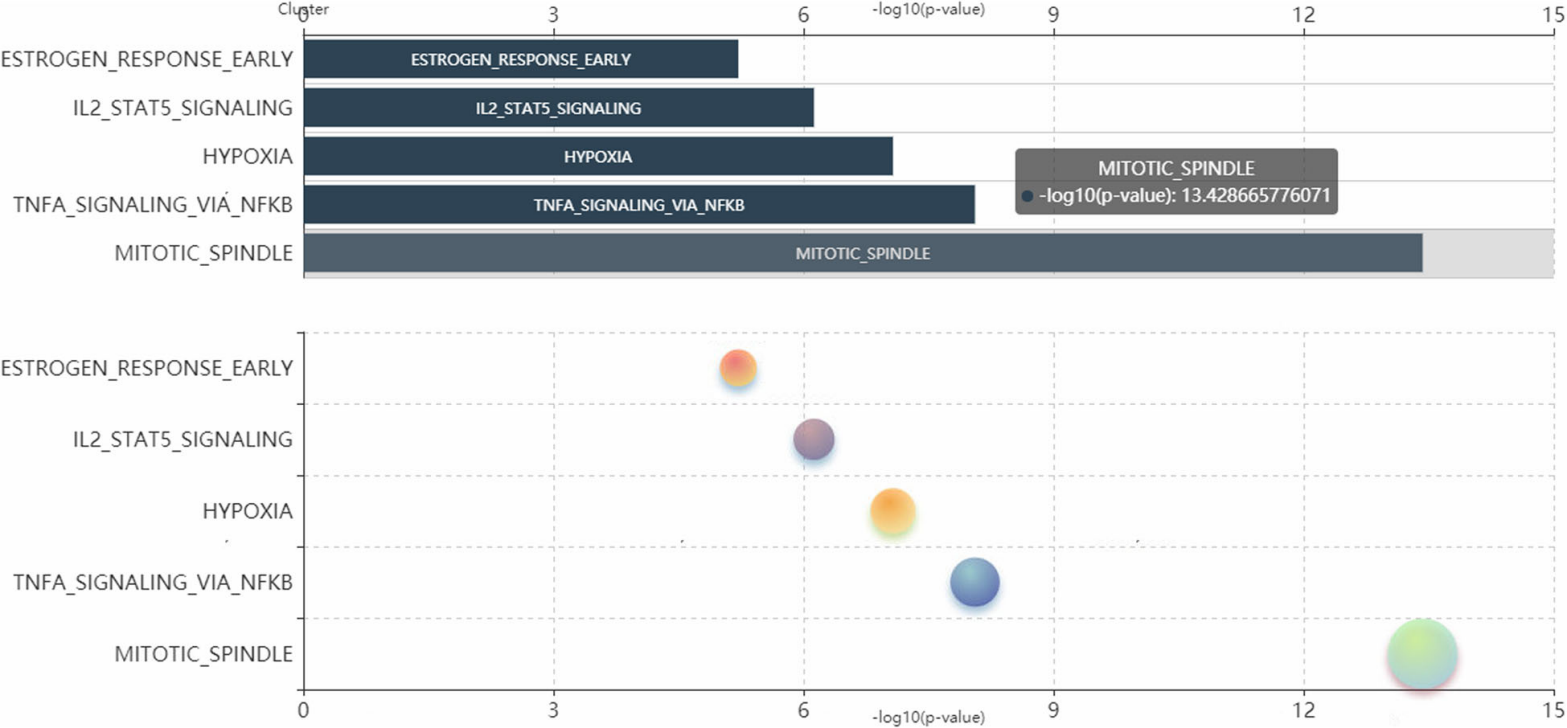
An example functional enrichment analysis results of hypermethylated genes

The pipeline also has been made available as the m6Acorr server, which could perform methylation profile correction based on the user-provided batch (experiment) assignment. If no batch is assigned, then the entire dataset will be treated as one experiment. In addition, users could assign experimental groups of samples (e.g., diseased and healthy). Thus, the hyper- (hypo-) methylated genes for each group can be derived on the basis of the Z-score by comparing the intra-group methylation level with the methylation level in other profiles. Moreover, their enriched functions can be analyzed on the basis of the curated gene set annotations in the m6Acorr server (Fig. 4).

## Conclusions

The current work focused on the existing laboratory bias in RNA m^6^A methylation profiles of public databases and developed m6Acorr, a pipeline for m^6^A profile correction based on quantile normalization and empirical Bayes batch regression methods. m6Acorr achieved the favorable results in real and artificial datasets. While the bias was eliminated by m6Acorr, the biological signals were well preserved after correction. The m6Acorr server could also be used to compare m^6^A profiles and conduct the functional analysis of hyper- (hypo-) methylated genes based on corrected methylation profiles. Overall, the m6Acorr server could be a useful tool for the correction and comparison of m^6^A methylation profiles.

## Supplementary information

**Additional file 1: Figure S1**. Comparison between intra-group correlation among SE/DC group and SC/DE group in mouse methylation dataset. **Figure S2.** Comparison between intra-group correlation among SE/DC group and SC/DE group under different quantiles. **Figure S3.** Comparison between intra-group correlation among SE/DC group and SC/DE group in the simulated dataset. **Figure S4.** Correlation curves between the m^6^A regulation breadth and various gene importance-related features. **Table S1.** Grid search of the parameters to fit the real world laboratory bias

## Data Availability

The datasets generated and/or analyzed during the current study are available in the MeT-DB v2.0 repository, http://www.xjtlu.edu.cn/metdb2.

## Abbreviations

CV: Coefficient of variation
IP: Immunoprecipitation
m^6^A: N^6^-methyladenosine
miRNA: MicroRNA
mRNA: Messenger RNA
PPI: Protein–protein interaction
SC/DE: Same cell but different experiments
SE/DC: Same experiment but different cells
