# Supplementary material for: m6Acorr: an online tool for the correction and comparison of m6A methylation profiles
Source: BMC Bioinformatics. 2020 Jan 29;21:31. doi: 10.1186/s12859-020-3380-6 (PMC6988237; doi:10.1186/s12859-020-3380-6)
Supplement: Supplementary file 1 — Additional file 1: Figure S1. Comparison between intra-group correlation among SE/DC group and SC/DE group in mouse methylation dataset. Figure S2. Comparison between intra-group correlation among SE/DC group and SC/DE group under different quantiles. Figure S3. Comparison between intra-group correlation among SE/DC group and SC/DE group in the simulated dataset. Figure S4. Correlation curves between the m6A regulation breadth and various gene importance-related features. Table S1. Grid search of the parameters to fit the real world laboratory bias [file 12859_2020_3380_MOESM1_ESM.doc]

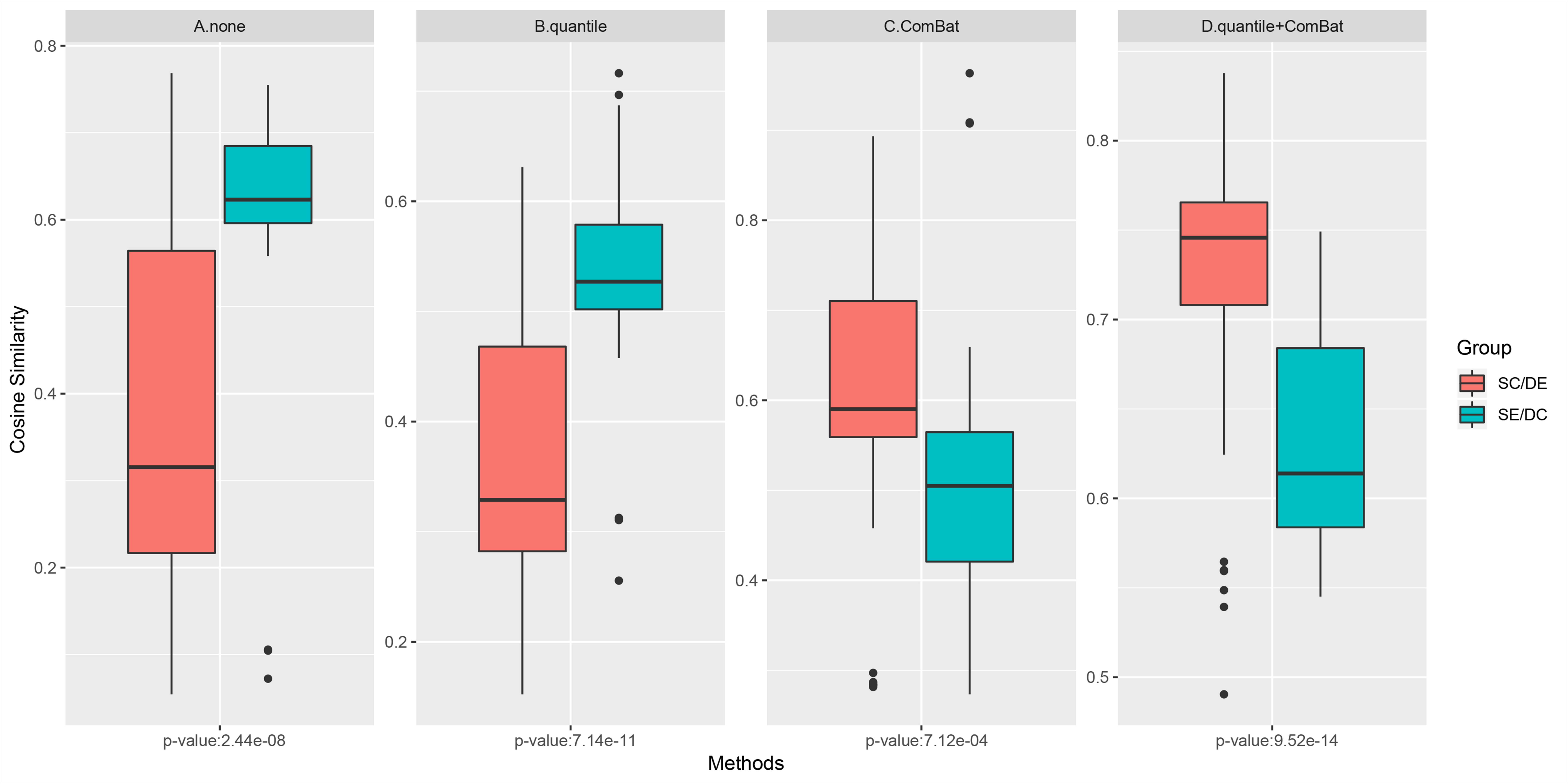


**Figure S1.  Comparison between intra-group correlation among SE/DC group and SC/DE group in mouse methylation dataset.** SE/DC, same experiment across different cell types; SC/DE, same cell type but different experiments. Intuitively, less biased methylation profiles should have significantly higher correlation in SE/DC group than the SC/DE group, which can be achieved by combing ComBat method and quantile normalization. The p-values were obtained by t-test.


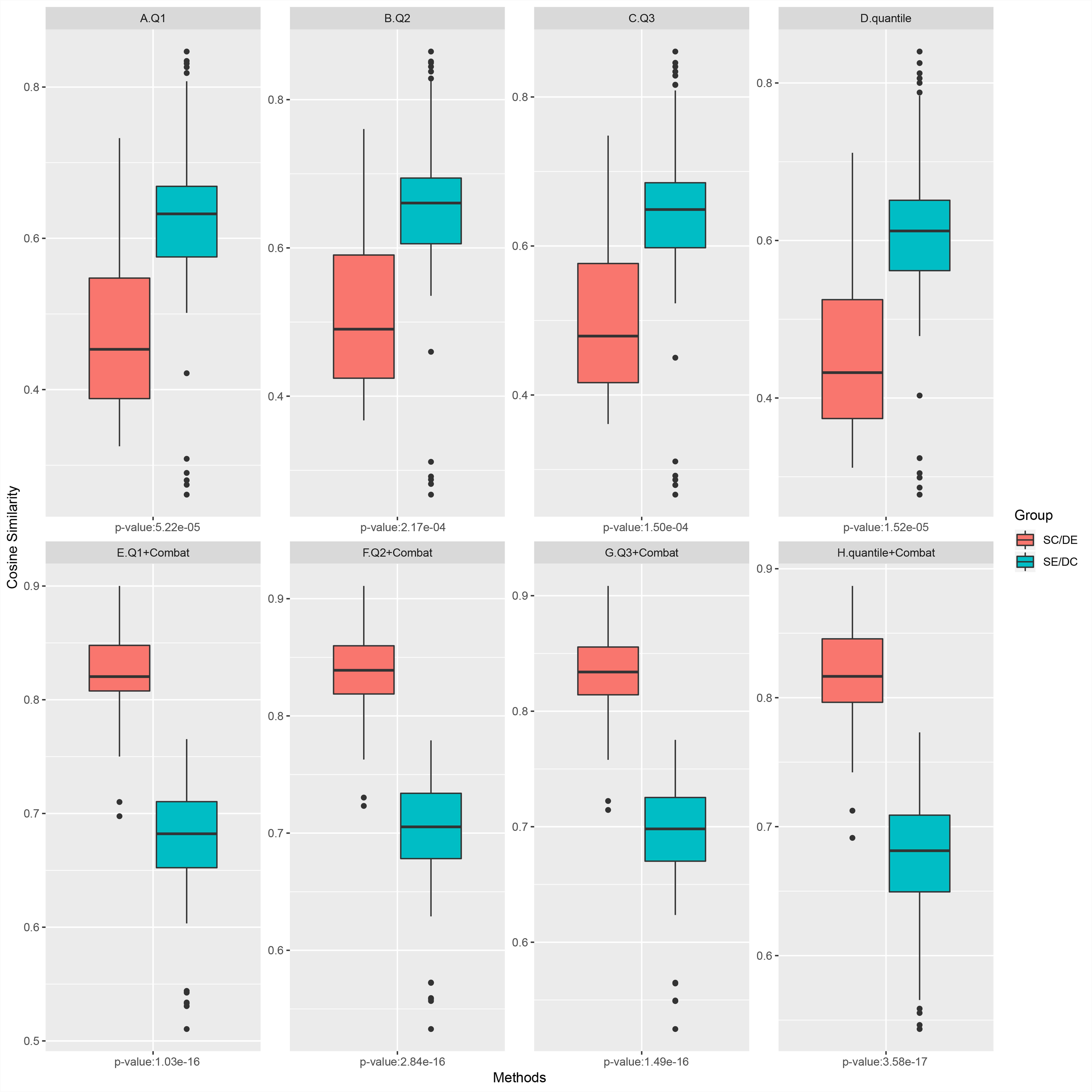


**Figure S2.  Comparison between intra-group correlation among SE/DC group and SC/DE group under different quantiles.** Q1, Q2, Q3 quantiles and the standard quantile normalization obtained similar results and we finally chose the mainstream practice i.e. standard quantile normalization as part of our pipeline. P-values were obtained by t-test.


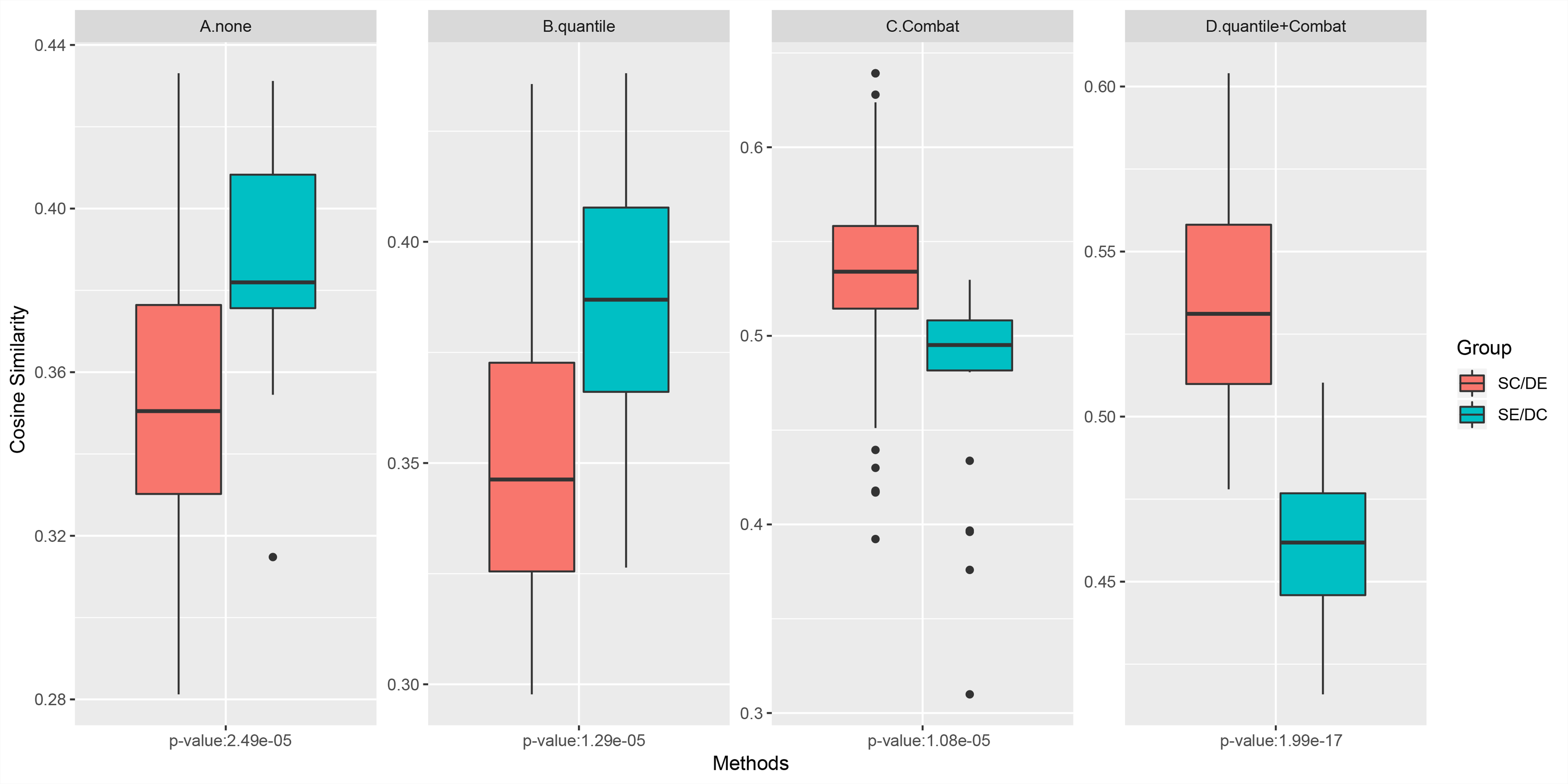


**Figure S3.  Comparison between intra-group correlation among SE/DC group and SC/DE group in the simulated dataset.** Obviously, after correction by the pipeline combining quantile normalization and Combat batch regression methods, the unwanted higher correlation (SE/DC over SC/DE, p = 2.49e-05) has been reversed (SE/DC under SC/DE, P = 1.99e-17). The p-values were obtained by t-test.


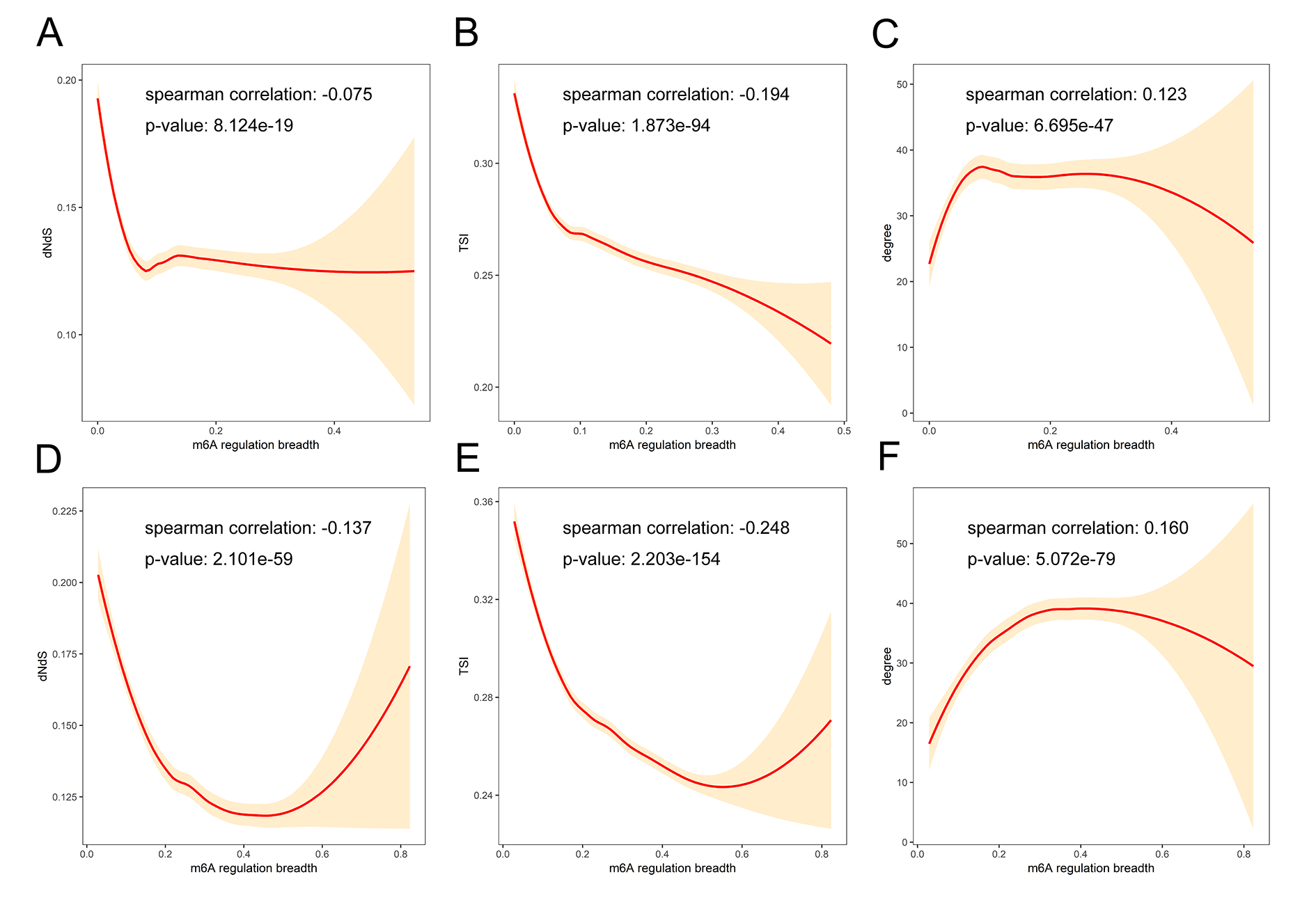


**Figure S4. Correlation curves between the m^6^A regulation breadth and various gene importance-related features.** The correlations before (A-C) and after (D-F) correction are shown. The fitted curves are plotted by LOESS smoothing. The shade indicates the confidence interval. The p-values were obtained by Spearman correlation test. **A.** Correlation of m^6^A regulation breadth profiles with dN/dS ratio, using the uncorrected profiles. **B.** Correlation of m^6^A regulation breadth profiles with PPI network, using the uncorrected profiles. **C.** Correlation of m^6^A regulation breadth profiles with tissue expression specificity, using the uncorrected profiles. **D.** Correlation of m^6^A regulation breadth profiles with dN/dS ratio, using the corrected profiles. **E.** Correlation of m^6^A regulation breadth profiles with PPI network, using the corrected profiles. **F.** Correlation of m^6^A regulation breadth profiles with tissue expression specificity, using the corrected profiles.

**Table S1. Grid search of the parameters to fit the real world laboratory** bias.

| batch.facLoc | batch.facScale | P-value |
| --- | --- | --- |
| real-world | real-world | 7.08E-06 |
| 0.1 | 0.1 | 7.34E-01 |
| 0.1 | 0.2 | 1.45E-01 |
| 0.1 | 0.3 | 9.65E-04 |
| 0.2 | 0.1 | 7.81E-01 |
| 0.2 | 0.2 | 3.25E-02 |
| 0.2 | 0.3 | 5.49E-05 |
| 0.2 | 0.4 | 1.61E-14 |
| 0.3 | 0.1 | 1.02E-03 |
| 0.3 | 0.2 | 2.49E-05 |
| 0.3 | 0.3 | 6.66E-14 |
| 0.3 | 0.4 | 4.93E-14 |
| 0.4 | 0.1 | 3.79E-07 |
| 0.4 | 0.2 | 5.42E-09 |
| 0.4 | 0.3 | 3.66E-16 |
| 0.4 | 0.4 | 1.44E-19 |
